# JAG1/Notch Pathway Inhibition Induces Ferroptosis and Promotes Cataractogenesis

**DOI:** 10.3390/ijms26010307

**Published:** 2025-01-01

**Authors:** Yan Ni, Liangping Liu, Fanying Jiang, Mingxing Wu, Yingyan Qin

**Affiliations:** 1 State Key Laboratory of Ophthalmology, Zhongshan Ophthalmic Center, Sun Yat-sen University, Guangdong Provincial Key Laboratory of Ophthalmology and Visual Science, Guangzhou 510060, China; niyan@mail2.sysu.edu.cn (Y.N.) liulp2@mail.sysu.edu.cn (L.L.); jiangfy3@mail2.sysu.edu.cn (F.J.); 2 Guangdong Provincial Clinical Research Center for Ocular Diseases, Guangzhou 510033, China

**Keywords:** Notch, ferroptosis, age-related cataract, lens epithelial cells

## Abstract

Cataracts remain the leading cause of visual impairment worldwide, yet the underlying molecular mechanisms, particularly in age-related cataracts (ARCs), are not fully understood. The Notch signaling pathway, known for its critical role in various degenerative diseases, may also contribute to ARC pathogenesis, although its specific involvement is unclear. This study investigates the role of Notch signaling in regulating ferroptosis in lens epithelial cells (LECs) and its impact on ARC progression. RNA sequencing of anterior lens capsule samples from ARC patients revealed a significant downregulation of Notch signaling, coupled with an upregulation of ferroptosis-related genes. Notch1 expression decreased, while ferroptosis markers increased in an age-dependent manner. In vitro, upregulation of Notch signaling alleviated ferroptosis by decreasing ferritin heavy chain 1 (FTH1) and p53 levels while enhancing the expression of nuclear factor erythroid 2-related factor 2 (Nrf2), glutathione peroxidase 4 (GPX4), and solute carrier family 7 member 11 (SLC7A11). Conversely, inhibition of Notch signaling exacerbated ferroptosis, as evidenced by reduced Nrf2, GPX4, and SLC7A11 expression. These findings suggest that downregulation of Notch signaling promotes ferroptosis in LECs by impairing the Nrf2/GPX4 antioxidant pathway, thereby contributing to ARC development. This study offers new insights into ARC pathogenesis and highlights the Notch signaling pathway as a potential therapeutic target for preventing or mitigating ARC progression.

## 1. Introduction

Cataract, characterized by lens opacity, stands as the leading cause of visual impairment globally, with age-related cataract (ARC) accounting for the majority of cases [1]. With an aging population worldwide, the incidence of ARC continues to rise, posing significant public health challenges. Currently, surgery is the most preferred effective treatment, improving visual conditions but incurring substantial costs and potential intraoperative and postoperative complications [2]. This highlights the pressing need for a deeper understanding of cataract pathogenesis and the identification of effective pharmacological treatment targets.

The precise pathogenesis of ARC remains incompletely understood; however, it is closely associated with aging and oxidative stress in lens epithelial cells (LECs) [3]. Nutrients and antioxidants in the aqueous humor play a crucial role in maintaining redox homeostasis within LECs to preserve lens transparency. Aging leads to an accumulation of reactive oxygen species (ROS) and a reduction in antioxidant defenses including glutathione (GSH) and glutathione peroxidase (GPX), contributing to cataract formation [4]. Disruption of GSH homeostasis affects the balance between ascorbic acid and dehydroascorbic acid, impacting iron (Fe^3+^) to iron (Fe^2+^) conversion through the Fenton reaction. Elevated levels of reactive iron (Fe^2+^) and copper (Cu^2+^) have been observed in cataractous lenses [5]. Additionally, increased ROS and impaired antioxidant defenses intensify lipid peroxidation, a process that accelerates with lens aging [6].

Ferroptosis, an iron-dependent form of programmed cell death [7], is characterized by intracellular iron accumulation, heightened lipid peroxidation, increased ROS, and reduced GSH and GPX levels [8]. Recent research has linked ferroptosis to various ocular pathologies [9], including cataracts. Ferroptosis can be induced in human and mouse LECs using low doses of cystine/glutamate reverse transporter inhibitors and GPX4 inhibitors. Furthermore, downregulation of ferroptosis-associated genes via solute carrier family 7 member 11 (SLC7A11), solute carrier family 3 member 2 (SLC3A2), and solute carrier family 40 member 1 (SLC40A1) in aging LEC cell lines (FHL124) suggests a role for ferroptosis in ARC development [10].

The Notch signaling pathway, a highly conserved mechanism that regulates cell differentiation, proliferation, and apoptosis, has been implicated in various degenerative conditions [11,12,13,14,15]. A previous study suggested that downregulation of Notch1 signaling can lead to elevated ROS levels and reduced GSH in cardiomyocytes [16]. Notably, Jagged1 (JAG1), a key ligand in the Notch signaling pathway, plays a crucial role in activating Notch1 [17,18], which in turn directly activates nuclear factor erythroid 2-related factor 2 (Nrf2), a key transcription factor in antioxidant defense. In response to oxidative stress, Nrf2 promotes the transcription of antioxidant enzymes such as GPX4 [19]. The suppression of GPX4 activity and depletion of GSH are recognized contributors to the process of ferroptosis [20,21].

However, the role of the Notch signaling pathway in the pathogenesis of cataracts, particularly ARC, has not been thoroughly studied. Research studies have been conducted on each of these individual components—oxidative stress, ferroptosis, and Notch signaling—but no study has comprehensively explored the intersection between these pathways in the context of ARC. We hypothesize that the dysregulation of Notch signaling impairs the cellular antioxidant defense system, including Nrf2 and GPX4, thereby promoting ferroptosis and contributing to cataract progression. By identifying the molecular connections between Notch signaling, cellular antioxidant pathways, and ferroptosis, we hope to uncover novel therapeutic targets for the prevention and management of ARC.

## 2. Results

### 2.1. The Role of Notch Signaling Pathway in ARC Formation

**Figure 1:**
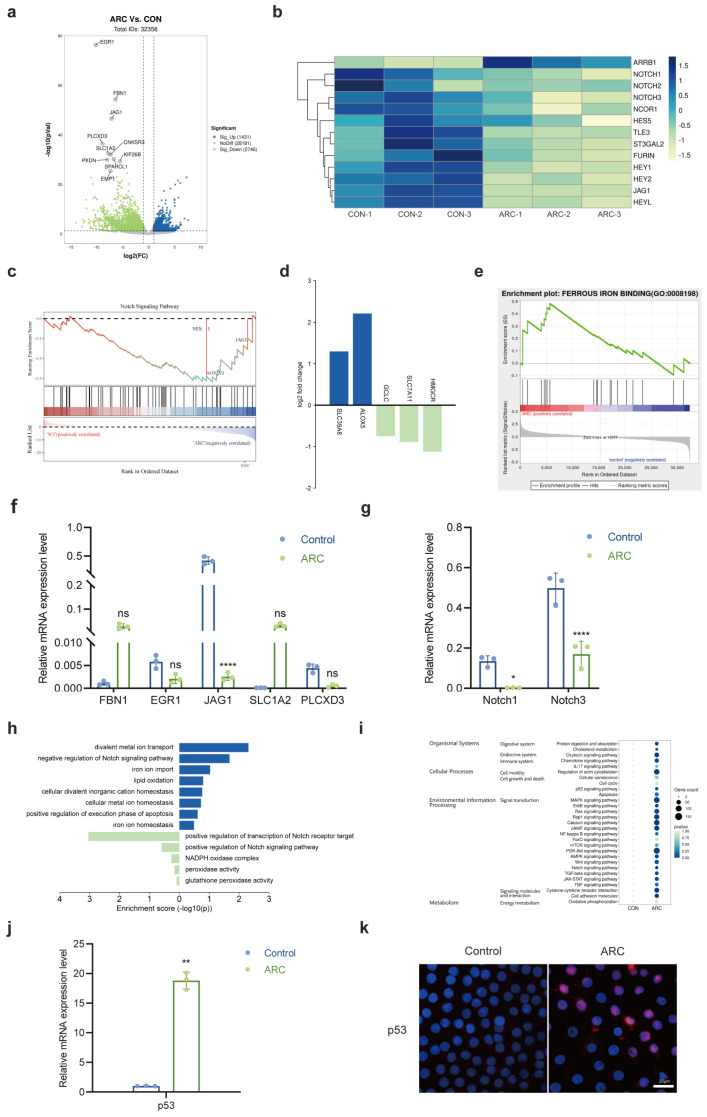
Transcriptomic and functional pathway analysis of ARC. (**a**) Volcano plots showing differentially expressed genes (DEGs) between control and ARC groups in −log10 scale and fold change in log_2_ scale. Significantly up- and downregulated genes (*p*-value < 0.05, absolute fold change > 2) are marked in blue and green, respectively, as indicated. Non-significantly regulated genes are marked in gray. (**b**) Heatmap showing the DEGs related to Notch signaling pathway. (**c**) Gene set enrichment analysis (GSEA) revealing the downregulation of Notch signaling pathway in ARC. (**d**) Bar graph of ferroptosis-related gene expression with fold change in log2 scale. (**e**) GSEA revealing the upregulation of ferroptosis-related pathways in ARC. (**f**) Downregulated mRNA levels of JAG1 in the ARC anterior lens capsules. (**g**) Downregulated mRNA levels of Notch1 and Notch3 in the ARC anterior lens capsules. (**h**) Bar graph of enriched terms showing Gene Ontology (GO) biological processes enrichment analysis of upregulated genes (right panel) and downregulated genes (left panel). (**i**) Dot plot for Kyoto Encyclopedia of Genes and Genomes (KEGG) enrichment results of DEGs shared between control and ARC groups. (**j**) Relative mRNA expression of p53 in the control and ARC groups. The relative level of mRNA was calculated using the 2^−ΔΔCT^ method. GAPDH served as loading control. (**k**) Representative immunofluorescence staining images of p53 (red) in anterior lens capsules. DAPI (blue) shows cell nuclei. Scale bars: 20 μm. Data are shown as mean ± SEM from three independent experiments. * *p* < 0.05, ** *p* < 0.01, **** *p* < 0.0001, ns, not significant.

To assess the comprehensive alterations in gene expression associated with ARC, we performed RNA sequencing analysis on anterior lens capsule samples obtained from ARC patients during surgery. Anterior lens capsule samples from donors with clear lenses served as the control group. Each sample was sequenced individually. Volcano plots showed the differentially expressed genes (DEGs) between the control and ARC groups. Among the top ten significantly expressed genes, JAG1 was found to be the third most significantly differentially expressed (Figure 1a). Given that JAG1 is a Notch ligand, we proceeded to analyze the Notch pathway components, observing significant downregulation of Notch receptors (Notch1, Notch2, Notch3) and downstream genes (Hes5, Hey1, Hey2, HeyL) (Figure 1b). Gene set enrichment analysis (GSEA) further verified the downregulation of the Notch signaling pathway (Figure 1c). Furthermore, we identified significant upregulation of ferroptosis-promoting genes (SLC39A8, ALOX5) and downregulation of ferroptosis-inhibiting genes (GCLC, SLC7A11, HMGCR) (Figure 1d). GSEA also corroborated the upregulation of the ferrous iron binding pathway in ARC (Figure 1e), which is consistent with the known role of iron metabolism in ferroptosis. This pathway is involved in the regulation of cellular iron levels, and its upregulation could contribute to iron overload, a key factor in the initiation and progression of ferroptosis, thus supporting the association between altered iron homeostasis and ferroptosis in ARC [22,23]. The results of RNA-seq were validated using qRT-PCR, which confirmed the significant downregulation of JAG1, Notch1, and Notch3 expression (Figure 1f,g). In addition, Gene Ontology (GO) enrichment analysis revealed upregulation of divalent metal ion transport, iron ion transport, lipid peroxidation, and cell senescence, as well as downregulation of the Notch signaling pathway (Figure 1h). Furthermore, a Kyoto Encyclopedia of Genes and Genomes (KEGG) analysis identified enrichment of terms related to p53, Notch, oxidative phosphorylation, cell cycle, inflammation, and the apoptosis pathway (Figure 1i). The RNA-seq results were validated through qRT-PCR and immunofluorescence, showing a significant increase in both p53 mRNA (Figure 1j) and protein levels (Figure 1k) in patients with ARC compared to the control group.

**Figure 2:**
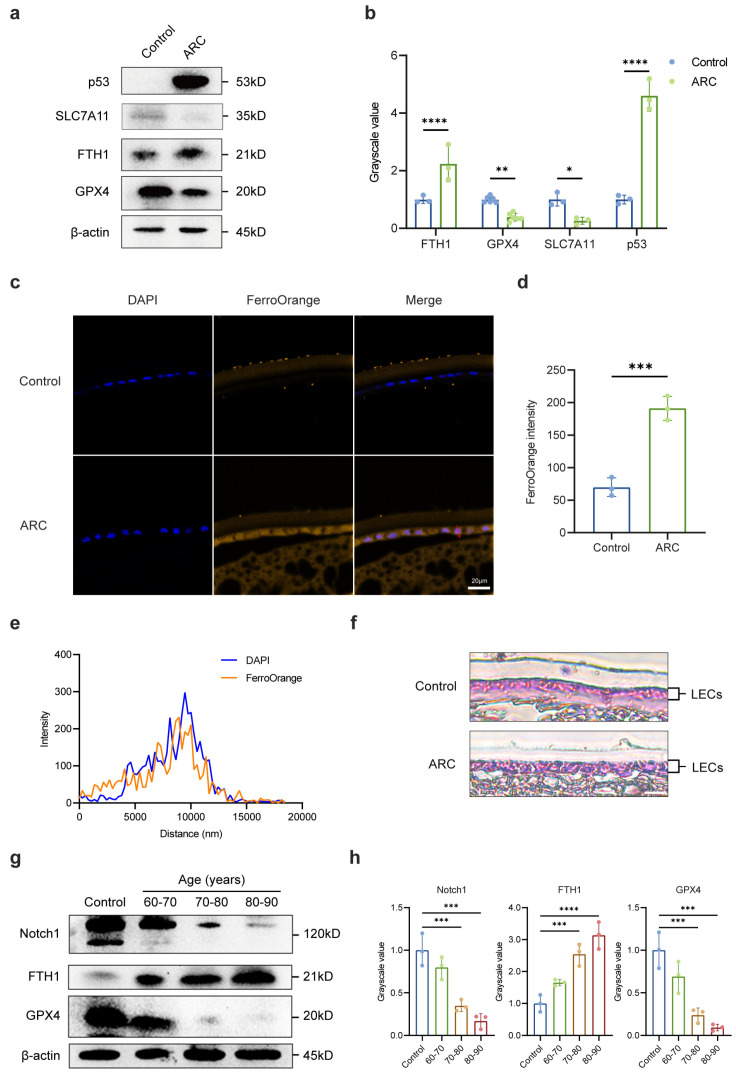
Evidence of oxidative damage-induced ferroptosis in LECs of ARC patients. (**a**,**b**) Western blot and densitometry analysis of FTH1, GPX4, SLC7A11, and p53 in anterior lens capsules, β-actin acted as internal controls. (**c**,**d**) Representative images and fluorescence intensity of FerroOrange staining in lens capsule sections. DAPI (blue) shows cell nuclei. Scale bars: 20 μm. (**e**) Colocation analysis of FerroOrange and DAPI in lens capsule sections from ARC patients along the red arrow indicated in panel c. (**f**) Representative images of Lillie staining for Fe^2+^ in lens capsule sections from the control and ARC groups. Fe^2+^ is stained blue, while nuclei are stained red. Scale bars: 5 μm. (**g**,**h**) Western blot and densitometry analysis of FTH1, GPX4, and Notch1 in anterior lens capsule samples from ARC patients aged 60–90 years, with donor lenses (mean age approximately 52.3 years) serving as controls. β-actin was used as the internal control. Data are shown as mean ± SEM from three independent experiments. * *p* < 0.05, ** *p* < 0.01, *** *p* < 0.001, **** *p* < 0.0001.

RNA-seq analysis tentatively confirmed the presence of ferroptosis in the anterior lens capsules from ARC patients. We further aimed to verify the occurrence of ferroptosis within the LECs of ARC. We assessed the levels of key antioxidant regulators, including ferritin heavy chain 1 (FTH1), solute carrier family 7 member 11 (SLC7A11, also termed xCT), glutathione peroxidase 4 (GPX4) and the ferroptosis-promoting gene p53 [24]. Comparative analysis showed a significant upregulation of FTH1 and p53 expression in the LECs of ARC patients alongside a downregulation of SLC7A11 as compared to the control group (Figure 2a,b). Moreover, lens sections stained with FerroOrange [25] demonstrated an accumulation of excessive Fe^2+^ in the LECs of ARC patients, as demonstrated by increased orange fluorescence (Figure 2c–e, Supplementary Figure S1). Additionally, Lillie ferrous staining revealed an augmented Fe^2+^ content in the cytoplasm of LECs in the ARC group compared to the control group, providing crucial evidence of ferroptosis (Figure 2f). Notably, we observed that advancing age was associated with a significant increase in FTH1 expression in the LECs of cataract patients, while GPX4 expression showed a corresponding decrease. Additionally, Notch1 expression declined in an age-dependent manner (Figure 2g,h). Together, these findings provide strong evidence for the occurrence of oxidative damage-induced ferroptosis in the LECs of ARC patients and suggest a possible role for the Notch signaling pathway in modulating antioxidant responses during cataract development.

### 2.2. Downregulation of Notch Signaling Pathway in Oxidative Stress-Treated LECs

**Figure 3:**
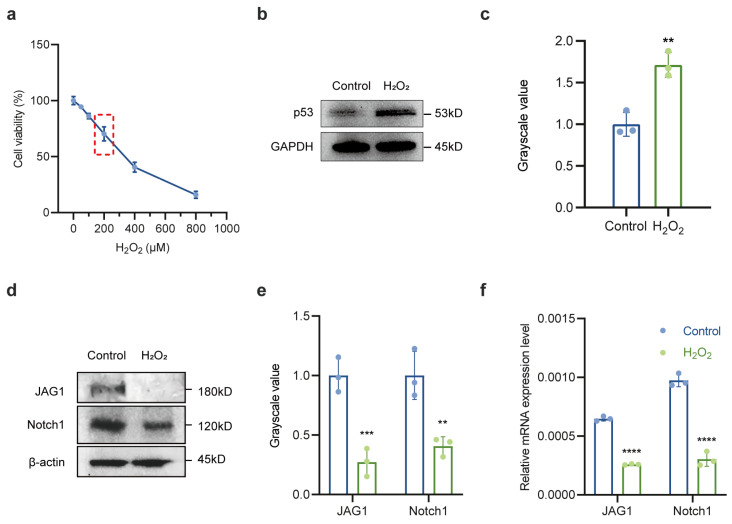
Downregulation of Notch signaling pathway in oxidative stress-treated LECs. (**a**) LECs were treated with varying concentrations of H_2_O_2_ for 24 h, and cell viability was assessed using a CCK-8 assay. The cells were divided into two groups: the control group (PBS, 24 h) and the H_2_O_2_ group (200 μM H_2_O_2_, 24 h). The red dashed box highlights the 200 μM H_2_O_2_; concentration range, selected for subsequent experiments due to its significant impact on cell viability. (**b**,**c**) Western blot and densitometry analysis of p53 in the control group and H_2_O_2_ group; GAPDH acted as internal control. (**d**,**e**) Western blot and densitometry analysis of JAG1 and Notch1 in the control group and H_2_O_2_ group; β-actin acted as internal control. (**f**) Relative mRNA expression of JAG1 and Notch1 in the control group and H_2_O_2_ group. The relative level of mRNA was calculated using the 2^−ΔΔCT^ method. GAPDH served as loading control. Data are shown as mean ± SEM from three independent experiments. ** *p* < 0.01, *** *p* < 0.001, **** *p* < 0.0001.

Given that oxidative stress is widely acknowledged as the leading cause of ARC, we created an in vitro cell culture model under oxidative stress conditions utilizing varying concentrations of H_2_O_2_. Findings from a CCK-8 assay demonstrated a dose-dependent decrease in cell viability of LECs following H_2_O_2_ treatment, with a reduction of approximately 30% observed after exposure to 200 μM H_2_O_2_ for 24 h (Figure 3a). Consequently, the 200 μM H_2_O_2_ concentration was selected for subsequent experiments. In comparison to the control group, p53 protein expression levels were significantly elevated in the H_2_O_2_ treatment group (Figure 3b,c). Additionally, the protein expression of the Notch signaling pathway ligand JAG1 and the receptor Notch1 (Figure 3d,e) as well as their corresponding mRNA levels (Figure 3f) were notably decreased. These results further corroborated the findings from the anterior lens capsule samples, indicating that Notch signaling was inhibited under oxidative stress during cataract formation.

### 2.3. Regulation of Notch Signaling Pathway Affected Susceptibility of LECs to Oxidative Stress

**Figure 4:**
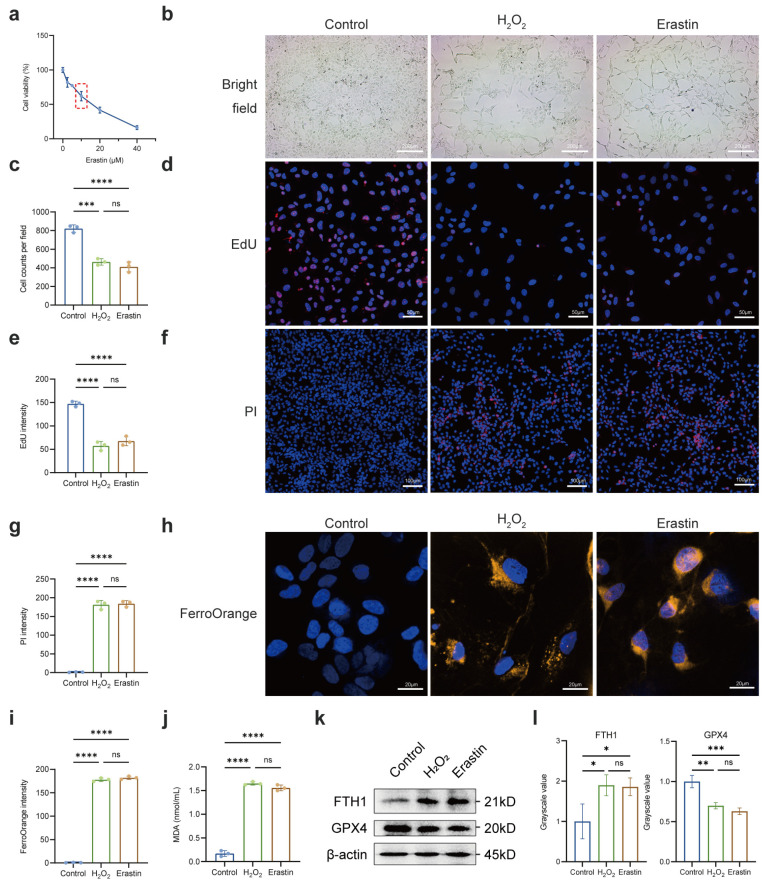
Ferroptosis occurred in oxidative stress-treated LECs. (**a**) LECs were treated with varying concentrations of Erastin for 24 h, and cell viability was assessed using a CCK-8 assay (10 μM Erastin, 24 h). The red dashed box highlights the 10 μM Erastin concentration range, selected for subsequent experiments due to its significant impact on cell viability. (**b**,**c**) Representative microscope images and ratio of cell number of control, H_2_O_2_, and Erastin groups. Scale bars: 200 μm. (**d**,**e**) Representative immunofluorescence images of EdU incorporation and quantification of the percentage of EdU-positive cells in control, H_2_O_2_, and Erastin groups. Scale bar: 50 μm. (**f**,**g**) Representative immunofluorescence images of PI incorporation and quantification of the percentage of PI-positive cells in control, H_2_O_2_, and Erastin groups. Scale bar: 100 μm. (**h**,**i**) FerroOrange staining and fluorescence intensity for Fe^2+^ of control, H_2_O_2_, and Erastin groups. Scale bars: 20 μm. (**j**) MDA levels in control, H_2_O_2_, and Erastin groups. (**k**,**l**) Western blot and densitometry analysis of FTH1 and GPX4 in control, H_2_O_2_, and Erastin groups; β-actin acted as internal control. Data are shown as mean ± SEM from three independent experiments. * *p* < 0.05, ** *p* < 0.01, *** *p* < 0.001, **** *p* < 0.0001, ns, not significant.

Previous studies have demonstrated a link between oxidative stress, increased ROS levels, and the initiation of lipid peroxidation, a key feature of ferroptosis, which can contribute to cellular damage, particularly to the cell membranes [26]. Therefore, we aimed to investigate the presence of oxidative damage-induced ferroptosis in LECs following treatment with H_2_O_2_. Initially, LECs were subjected to H_2_O_2_ and Erastin (utilized as an inducer of ferroptosis) [27]. CCK-8 assay indicated that 10 μM of Erastin was a suitable treatment concentration for subsequent experiments (Figure 4a–c). Our results revealed a substantial decrease in the percentage of EdU-positive cells following both H_2_O_2_ and Erastin treatments, signifying a decline in the proliferative capacity of LECs (Figure 4d,e). Additionally, the increased uptake of propidium iodide (PI) indicated compromised membrane integrity [28] (Figure 4f,g). FerroOrange staining of LECs demonstrated an accumulation of excessive Fe^2+^ within H_2_O_2_ and Erastin-treated cells (Figure 4h,i). Furthermore, elevated levels of lipid oxidative stress (LOS) were observed in LECs treated with H_2_O_2_ or Erastin, as evidenced by a significant accumulation of malondialdehyde (MDA), a common byproduct (Figure 4j). Notably, protein expression changes observed in the H_2_O_2_ group were consistent with those recorded in the Erastin group (Figure 4k,l). In summary, these findings affirmed the presence of ferroptosis in an H_2_O_2_-induced in vitro model of ARC.

**Figure 5:**
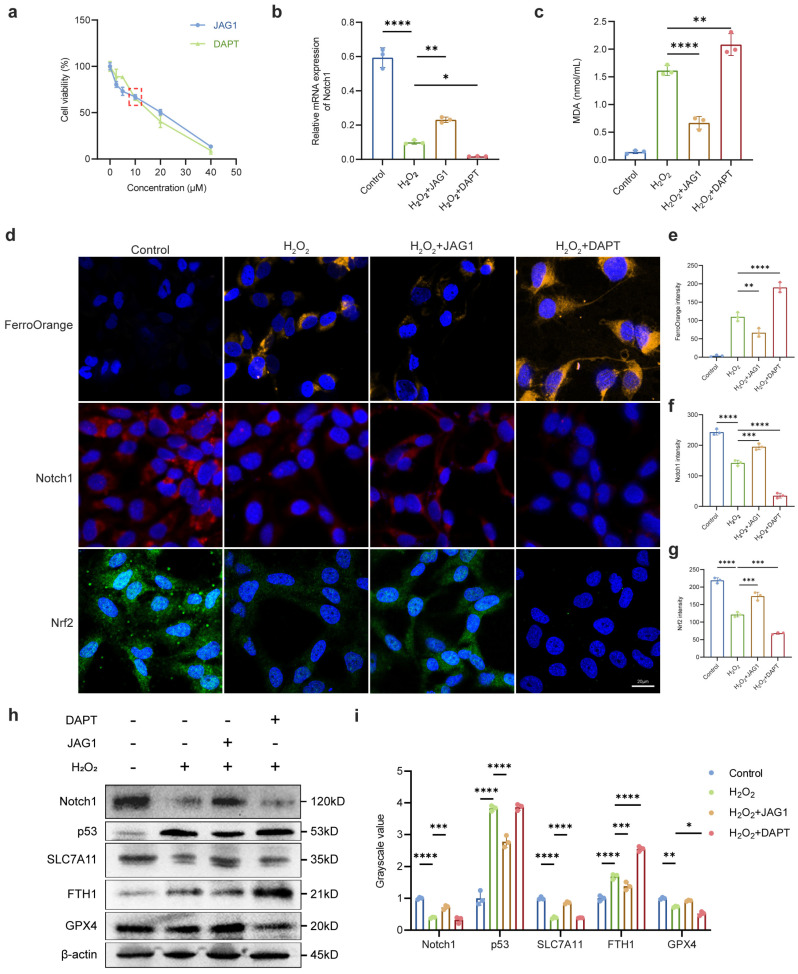
Regulation of Notch signaling pathway affected the susceptibility of LECs to oxidative stress. (**a**) LECs were treated with varying concentrations of JAG1 or DAPT for 24 h, and cell viability was assessed using a CCK-8 assay (10 μM JAG1, 24 h; 10 μM DAPT, 24 h). The red dashed box highlights the 10 μM concentrations of JAG1 and DAPT, selected for subsequent experiments due to their significant impact on cell viability. (**b**) Up- and downregulation of Notch signaling pathway was achieved by activator (JAG1) and inhibitor (DAPT), and qRT-PCR was used to verify the mRNA level of Notch1. GAPDH acted as internal control. (**c**) MDA level of control, H_2_O_2_, H_2_O_2_+JAG1, and H_2_O_2_+DAPT groups. (**d**) Representative images of FerroOrange and immunofluorescence staining of Nrf2 (green) and Notch1 (red) in control, H_2_O_2_, H_2_O_2_+JAG1, and H_2_O_2_+DAPT group. DAPI (blue) shows cell nuclei. Scale bars: 20 μm. (**e**–**g**) Fluorescence intensity for Fe^2+^, Nrf2, and Notch1 of control, H_2_O_2_, H_2_O_2_+JAG1, and H_2_O_2_+DAPT groups. (**h**,**i**) Western blot and densitometry analysis of GPX4, FTH1, SLC7A11, p53, and Notch1 in control, H_2_O_2_, H_2_O_2_+JAG1, and H_2_O_2_+DAPT groups; β-actin acted as internal control. Data are shown as mean ± SEM from three independent experiments. * *p* < 0.05, ** *p* < 0.01, *** *p* < 0.001, **** *p* < 0.0001.

To investigate the regulatory role of the Notch signaling pathway on oxidative damage-induced ferroptosis, we modulated Notch expression in vitro to assess changes in susceptibility to ferroptosis of LECs. Using JAG1 and DAPT to, respectively, promote and inhibit Notch expression in LECs, suitable treatment concentrations were determined through CCK-8 assays (Figure 5a). The modulatory effects were assessed through qRT-PCR. The outcomes indicated that compared to the H_2_O_2_ group, the H_2_O_2_ + JAG1 group exhibited a significant increase in RNA expression levels of Notch, while the H_2_O_2_+DAPT group displayed significantly decreased Notch expression (Figure 5b). We then revealed that alterations in Notch expression led to corresponding reductions or increases in intracellular LOS levels induced by H_2_O_2_ (Figure 5c). FerroOrange staining confirmed that upregulation of Notch substantially reduced Fe^2+^ accumulation in LECs, contrasting with the opposite effect observed with Notch downregulation. Immunofluorescence results highlighted that Notch inhibition significantly reduced Nrf2 levels, the core transcription factor of the antioxidant system. The opposite trend was observed when Notch expression was upregulated (Figure 5d–g), aligning with the hypothesis that Notch signaling influences the antioxidant capacity of LECs. Compared to the H_2_O_2_ group, Western blot demonstrated that Notch inhibition heightened LECs’ susceptibility to ferroptosis following H_2_O_2_ exposure, as evidenced by reduced levels of GPX4 and SLC7A11. Conversely, Notch upregulation mitigated the extent of ferroptosis, illustrated by the elevation of GPX4 and SLC7A11 alongside the reduction of FTH1 and p53 (Figure 5h,i).

## 3. Discussion

**Figure 6:**
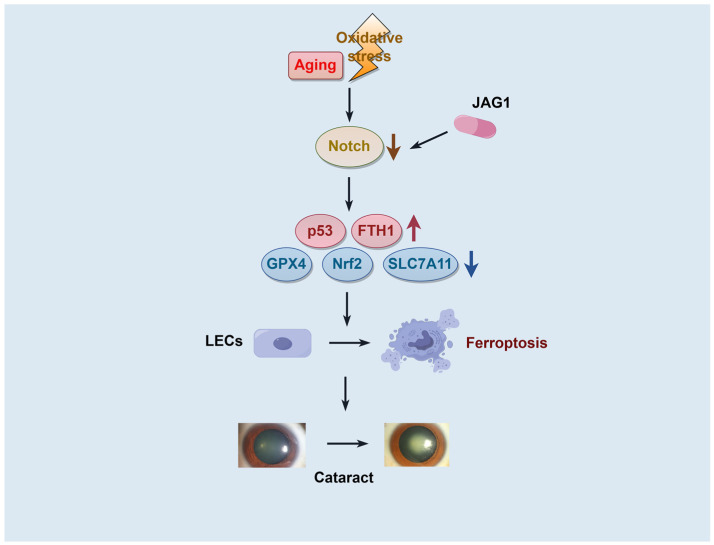
Schematic diagram of the role of Notch signaling in modulating ferroptosis and its contribution to cataract formation. In aging and oxidative stress conditions, downregulation of Notch signaling results in the suppression of the Nrf2/GPX4 antioxidant system. This suppression leads to increased cellular lipid peroxidation, elevated ROS levels, and iron accumulation, which promote ferroptosis in lens epithelial cells (LECs). Ferroptosis, as a manifestation of impaired antioxidant defense, accelerates cataractogenesis. Conversely, activation of the Notch pathway by JAG1 upregulates the Nrf2/GPX4 axis and enhances the expression of the ferroptosis-inhibiting gene SLC7A11, thereby protecting LECs from ferroptosis and mitigating cataract progression. This pathway underscores the critical role of Notch signaling in regulating oxidative stress and ferroptosis in the context of age-related cataracts. Created by Figdraw.

In this study, we first characterized the role of the JAG1/Notch pathway in modulating oxidative damage-induced ferroptosis in LECs and its contribution to ARC formation. We found that as the Notch signaling gradually decreases in an age-dependent manner, there is a corresponding weakening of the Nrf2/GPX4 antioxidant system and an enhancement of p53 signaling. This leads to elevated levels of lipid peroxidation and ROS in LECs. Additionally, excessive iron accumulation contributes to an increased incidence of ferroptosis, ultimately leading to the development of age-related cataracts (Figure 6).

Previous research has established the connection between ferroptosis and cataract formation, with lipid peroxidation and oxidative stress playing central roles. Ferroptosis occurs when intracellular iron accumulates and ROS levels rise, damaging cellular membranes and leading to the death of LECs [9]. This cellular damage ultimately results in lens opacity. Aging might exacerbate these ferroptotic processes as antioxidant defenses, such as GSH and GPX4, which help neutralize lipid peroxides and prevent ferroptosis, are depleted in aging LECs [10,29]. Indeed, our study confirms the significant role of ferroptosis in ARC by showing upregulation of ferroptosis-promoting genes (SLC39A8 [30], ALOX5 [31]) and downregulation of ferroptosis-inhibiting genes (GCLC [32], SLC7A11 [33], HMGCR [34]) in the LECs of ARC patients. We also found that the ferrous iron (Fe^2+^) binding pathway was activated and markers of lipid peroxidation were elevated in the LECs of cataractous lenses, further confirming the presence of ferroptosis. Importantly, our data show that susceptibility to ferroptosis increases with age, as evidenced by increased levels of FTH1 and p53 alongside decreased GPX4 levels, indicating that aging promotes ferroptosis in LECs, accelerating cataract formation. This finding is consistent with previous research, demonstrating that aging enhances ferroptosis susceptibility in liver tissues [35]. Collectively, these observations reinforce the hypothesis that oxidative damage-induced ferroptosis plays a crucial role in cataractogenesis, particularly in the aging population.

The Notch signaling pathway plays a fundamental role in influencing cell proliferation, differentiation, and death through the mediation of intercellular communication, thus impacting disease progression [36]. In recent years, accumulating evidence has linked the Notch pathway to various degenerative diseases [37]. For example, in aged human epidermis, the expression of its ligand JAG1 is significantly reduced compared to that in younger individuals [12]. Similarly, in bone tissue, transcriptome sequencing has shown a marked downregulation of the Notch pathway in elderly mice [13]. In vascular endothelial cells, low JAG1 expression has been associated with cellular senescence, leading to cardiovascular diseases [14]. Further, studies involving the transfer of Presenilin 1 (PSEN1) into mutant mice to activate gamma-secretase, and thereby the Notch pathway, have demonstrated improvements in memory impairment and alleviation of neurodegeneration [15], underscoring the critical role of Notch signaling in degenerative conditions.

While the role of Notch signaling in cataract formation is still under investigation, our study provides new insights by demonstrating that downregulation of Notch signaling in LECs plays a significant role in ARC development, suggesting a novel connection between Notch signaling and ferroptosis through the Nrf2/GPX4 antioxidant pathway and p53. Specifically, we observed significant reductions in JAG1 and Notch receptors (Notch1, Notch2, and Notch3) in the LECs of cataract patients. In addition, the expression of Notch1 was found to decrease in an age-dependent manner. And the downregulation of Notch pathway was accompanied by increased ferroptosis activity, demonstrated by elevated p53 expression, as validated by qRT-PCR and immunofluorescence analyses.

Both the Notch signaling pathway and ferroptosis have been implicated in the pathogenesis of age-related diseases [14,38]. This suggests the potential for an interactive mechanism between the Notch signaling pathway and ferroptosis. On one hand, the Notch signaling pathway has been reported to regulate the Nrf2/GPX4 axis, thereby reducing oxidative stress and ferroptosis. On the other hand, p53 is a well-known promoter of ferroptosis [39]. In cancer-related research, inhibition of the Notch signaling pathway has been shown to upregulate the expression of p53 [40,41]. In our study, we modulated Notch signaling in H_2_O_2_-treated LECs, using JAG1 to upregulate Notch and DAPT to inhibit it. Inhibition of the Notch pathway increased the cells’ susceptibility to ferroptosis, as indicated by reduced expression of Nrf2, GPX4, and SLC7A11. Conversely, upregulation of Notch signaling mitigated ferroptosis, evidenced by decreased FTH1 and p53 expression alongside increased Nrf2, GPX4, and SLC7A11 levels, which play a critical role in ferroptosis resistance. This highlights a direct crosstalk between Notch signaling and ferroptosis through the Nrf2/GPX4 axis and p53 regulation.

The increased FTH1 expression observed in our study is noteworthy, as FTH1 is generally considered a protective factor against ferroptosis by sequestering free iron. However, we interpret this upregulation as a compensatory response to the elevated oxidative stress and iron dysregulation present in ARC. FTH1 functions to mitigate iron-induced damage by binding excess iron, but in prolonged oxidative stress conditions, this protective mechanism may become overwhelmed. Similar findings have been reported in severe cataract patients [38], where elevated FTH1 levels reflect an adaptive response rather than a protective one.

To conclude, our study elucidated the role of the Notch pathway in modulating ferroptosis through the Nrf2/GPX4 axis and p53 in LECs, as well as its contribution to ARC formation. The downregulation of Notch signaling under oxidative stress leads to the suppression of the antioxidant system, and ferroptosis becomes one of the manifestations of this oxidative damage, ultimately contributing to cataract formation. Notably, a previous study from our group demonstrated that the Notch pathway contributes to anterior subcapsular cataract development [42], highlighting its broader relevance in cataract pathogenesis. Although further translational studies are needed, targeting the Notch pathway could represent a novel and effective therapeutic strategy to prevent cataract progression, particularly in ARC.

## 4. Materials and Methods

### 4.1. Ethics Approval and Consent to Participate

Subjects were recruited from the Zhongshan Ophthalmic Center of Sun Yat-sen University (Guangzhou, China) between September 2023 and June 2024. The study protocol received approval from the Ethical Review Committee of the Zhongshan Ophthalmic Center (2021KYPJ168-2). All procedures involving human subjects were conducted in accordance with the World Medical Association’s Declaration of Helsinki. Informed written consent was obtained from each participant prior to their inclusion in this study.

### 4.2. Collection of Human Lens Samples

Anterior capsule samples were collected from patients with ARC during cataract surgeries, with each sample measuring approximately 5 mm in diameter. All surgeries were performed by Dr. Mingxing Wu. Additionally, anterior capsule samples from transparent lenses were obtained from donor eyes through the Guangdong Eye Bank. Samples were harvested within 5 h postmortem and immediately transferred to ice for preservation. The samples intended for immunofluorescence were fixed immediately, while the remaining samples were stored frozen at −80 °C. Detailed information about the patients and donors can be found in Supplementary Tables S1 and S2.

### 4.3. Western Blot Assay

Selected samples were processed for lysis utilizing pre-cooled extraction buffer (MA0171, Meilunbio, Dalian, China) and subsequently centrifuged to obtain the supernatant. Following quantification of protein concentration with the BCA assay, sample extracts were supplemented with SDS-PAGE loading buffer and denatured at 100 °C for 10 min. These extracts were then separated via 12.5% gradient SDS-PAGE. Protein bands were transferred onto a polyvinylidene difluoride (PVDF) blotting membrane (Millipore, Billerica, MA, USA), and immunolabeling was carried out using primary and secondary antibodies. Immunoblot bands were visualized using an ECL chemiluminescence detection kit (Merck Millipore, Darmstadt, Germany). The intensity of the bands was quantified utilizing ImageJ software (version 1.53t, USA). The antibodies used are listed in Supplementary Table S3.

### 4.4. Quantitative Real-Time Polymerase Chain Reaction

Total RNA was extracted utilizing RNAiso reagent (TAKARA, Shiga, Japan), following the manufacturer’s provided protocol. Subsequently, the RNA samples were reverse-transcribed into complementary DNA (cDNA) using a PrimeScript™ RT Master Mix kit (TAKARA, Japan). For the analysis of relative gene expression, quantitative real-time fluorescence PCR (qRT-PCR) was performed using Taq Pro Universal SYBR qPCR Master Mix (Q712-02, Vazyme, Nanjing, China) and a Roche LightCycler 480 system (Roche, Basel, Switzerland), following the manufacturer’s instructions. The qPCR primer sequences utilized in this study are listed in Supplementary Table S4.

### 4.5. RNA Sequence Analysis

Total RNA was extracted from lens capsules using TRIzol reagent (RNAiso Plus, 9108; TaKaRa Bio, Inc., Shiga, Japan). RNA concentrations were measured with a Nanodrop 2000 (Thermo Fisher Scientific, Waltham, MA, USA), and the integrity and purity were assessed using an Agilent Fragment Analyzer 5400 (Agilent Technologies, Santa Clara, CA, USA). Only RNA samples with a total amount greater than 400 ng and an RNA integrity number (RIN) above 7 were used for analysis, with three biological replicates in each group. Sequencing libraries were prepared using a NEBNext Ultra™ RNA Library Prep Kit for Illumina (New England Biolabs Inc., Ipswich, MA, USA) and sequenced on an Illumina NovaSeq6000 platform after cluster generation. The reference genome index was constructed, and paired-end clean reads were aligned to the reference genome using HISAT2 (version 2.0.5). RNA-Seq data analysis was conducted through NovoMagic (magic.novogene.com) (accessed on 25 March 2024). Read counts for each gene were generated using FeatureCounts (version 1.5.0-p3), and differential gene expression analysis was performed using the DESeq2 R package (version 1.20.1). GO enrichment for differentially expressed genes (DEGs; *p* < 0.05) was conducted with the clusterProfiler R package. Data visualization and analysis were carried out using online tools (https://www.bioinformatics.com.cn/; https://www.omicstudio.cn/tool/) (accessed on 6 May 2024).

### 4.6. Cell Culture and Treatment

The immortalized human lens epithelial cell line SRA 01/04 was generously provided by Professor Fu Shang at the Laboratory of Nutrition and Vision Research (Boston, MA, USA). The cells were cultured in Dulbecco’s Modified Eagle’s Medium (DMEM; Gibco, Grand Island, NY, USA) supplemented with 10% fetal bovine serum (FBS; Gibco, USA). An oxidative stress model was induced using H_2_O_2_ treatment (200 µM) for 24 h in the absence of FBS. A total of 10 µM Erastin (MCE, Shanghai, China), 10 µM DAPT (MCE, China), and 10 µM JAG1 (MCE, China) each was used for the treatment of LECs for the indicated time period.

### 4.7. Immunofluorescence

The donor lenses were immersed in 4% paraformaldehyde (PFA) for fixation overnight. Dehydration with 25% sucrose was performed overnight, and the lenses were embedded in optimal cutting temperature compound (O.C.T) and sectioned into 10 μm samples by a Leica cryostat. The capsule sample was washed three times with 1x PBS and then carefully placed flat on a slide with the cells facing upward. Then, the capsules and cultured cells were fixed with pre-cooled 100% methanol for 15 min. For immunostaining, the samples were permeabilized and blocked using PBS containing 1.5% bovine serum albumin and 0.5% Triton for a duration of 1 h. Subsequently, the samples were incubated with primary and secondary antibody solution. The cell nuclei were stained with DAPI (Beyotime, Shanghai, China) for 5 min. Imaging was performed using a Zeiss LSM880 laser scanning confocal microscope (Zeiss, Oberkochen, Germany) with a 63× 1.4 NA oil objective. The zoom magnification is specified in the figure legend. Raw images were processed using Zen (Zeiss) software (version 3.8).

### 4.8. Lillie Staining

Lillie staining (Biotopped, Beijing, China) was utilized to analyze changes in ferrous iron within the frozen sections of samples. Sections were incubated in Lillie staining solution for 30 min, stained with nuclear fast red for 7 min, and then sealed with neutral gum. Images were taken using an inverted microscope.

### 4.9. FerroOrange

To assess intracellular Fe^2+^ levels, FerroOrange (CST, Danvers, MA, USA) staining was performed according to the manufacturer’s instructions. LECs were treated with the relevant chemicals, then stained with 1 μmol/L FerroOrange for 30 min at room temperature. Imaging was performed using a confocal fluorescence microscope. Fluorescence intensity was quantified using ImageJ software (version 1.53t).

### 4.10. LOS Detection

Lipid oxygen species levels in LECs were measured using a Malondialdehyde (MDA) Assay Kit (Biotopped, Beijing, China), following the manufacturer’s instructions.

### 4.11. Statistical Analysis

All experiments were independently repeated at least three times. Statistical analysis was performed using GraphPad Prism (version 9.0.0, San Diego, CA, USA). Unpaired t-tests were used to assess differences between two datasets. For comparisons involving multiple datasets, one-way analysis of variance (ANOVA) was conducted, followed by post hoc tests. The Shapiro–Wilk test was used to check data normality. Post hoc Fisher’s least significant difference (LSD) was applied to normally distributed data, while Tamhane’s T2 was used for non-normally distributed data. Statistical significance was set at *p* < 0.05.

## Data Availability

The data used in the current study are available from the corresponding author upon reasonable request.

## Supplementary Materials

The following supporting information can be downloaded at: https://www.mdpi.com/article/10.3390/ijms26010307/s1.
