# Supplementary material for: JAG1/Notch Pathway Inhibition Induces Ferroptosis and Promotes Cataractogenesis
Source: Int J Mol Sci. 2025 Jan 1;26(1):307. doi: 10.3390/ijms26010307 (PMC11719987; doi:10.3390/ijms26010307)

Supplementary Table S1: Information about the patients with ARC

No.	Gender	Age	Operation eye	
1	F	55	OD	RNA-seq
2	F	66	OS	RNA-seq
3	M	60	OS	RNA-seq
4	F	62	OD	
5	F	74	OS	
6	M	63	OD	
7	F	63	OD	
8	M	59	OS	
9	M	58	OD	
10	F	62	OS	
11	M	72	OD	
12	M	78	OS	
13	F	76	OD	
14	M	72	OD	
15	F	73	OS	
16	M	84	OD	
17	F	88	OS	
18	M	81	OS	
19	F	85	OD	
20	F	81	OD	
21	M	85	OS	

Supplementary Table S2. Basic details of clear lens donors

No.	Age(years)	Cause of death	Lens status	
1	50	F	Road injury	RNA-seq
2	52	M	Intracerebral hemorrhage	RNA-seq
3	55	F	Gastric cancer	RNA-seq
4	52	F	Intracerebral hemorrhage	
5	48	M	Road injury	
6	45	F	Intracerebral hemorrhage	
7	53	M	Lung cancer	
8	47	M	Road injury	
9	49	M	Road injury	

Supplementary Table S3. Antibodies used in the experiments

**Antibody**	**Source**	**Company**	**Catalog No.**	**Application (dilution)**	**Category**
p53	mouse	CST	2524	WB(1:1000); IF(1:100)	primary antibody
SLC7A11	rabbit	CST	12691	WB(1:1000)	primary antibody
GPX4	rabbit	CST	59735	WB(1:500)	primary antibody
FTH1	rabbit	CST	4393S	WB(1:500)	primary antibody
Nrf2	rabbit	CST	12721	IF(1:200)	primary antibody
Notch1	rabbit	CST	4380	IF(1:200)	primary antibody
β-actin	rabbit	Abcam	ab8227	WB(1:1000)	primary antibody
Anti-rabbit IgG, HRP-linked Antibody	goat	CST	7074	WB(1:2000)	secondary antibody
Anti-mouse IgG, HRP-linked Antibody	horse	CST	7076	WB(1:2000)	secondary antibody
Alexa 488-conjugated anti-mouse antibody	goat	Bioss	bs-0296G-AF 488	IF(1:250)	secondary antibody
Alexa 594-conjugated anti-rabbit antibody	goat	Bioss	bs-0295G-AF 594	IF(1:250)	secondary antibody

CST: Cell Signaling Technology, Danvers, MA, USA; Abcam: Abcam, Cambridge, MA, USA; WB: Western blot; IF: Immunofluorescence

Supplementary Table S4. Primers for qRT-PCR used in the experiments

**Gene**	**Forward Sequences**	**Reverse Sequences**
GAPDH	5′-GGAGCGAGATCCCTCCAAAAT- 3′	5′- GGCTGTTGTCATACTTCTCATGG- 3′
Notch1	5′- CGCTGACGGAGTACAAGTG- 3′	5′- GTAGGAGCCGACCTCGTTG- 3′
Notch3	5′- GTAGAGGGCATGGTGGAAGA- 3′	5′- AAGTGGTCCAACAGCAGCTT- 3′
JAG1	5'- TGCCAAGTGCCAGGAAGT- 3'	5'- GCCCCATCTGGTATCACACT- 3'
p53	5′- TGTTTCCTGACTCAGAGGGG-3′	5′- GAGCGTGCTTTCCACGAC-3′

Supplementary Figure S1. Representative images of FerroOrange staining in lens capsule sections of patients. DAPI (blue) shows cell nuclei. Scale bars: 20 µm.

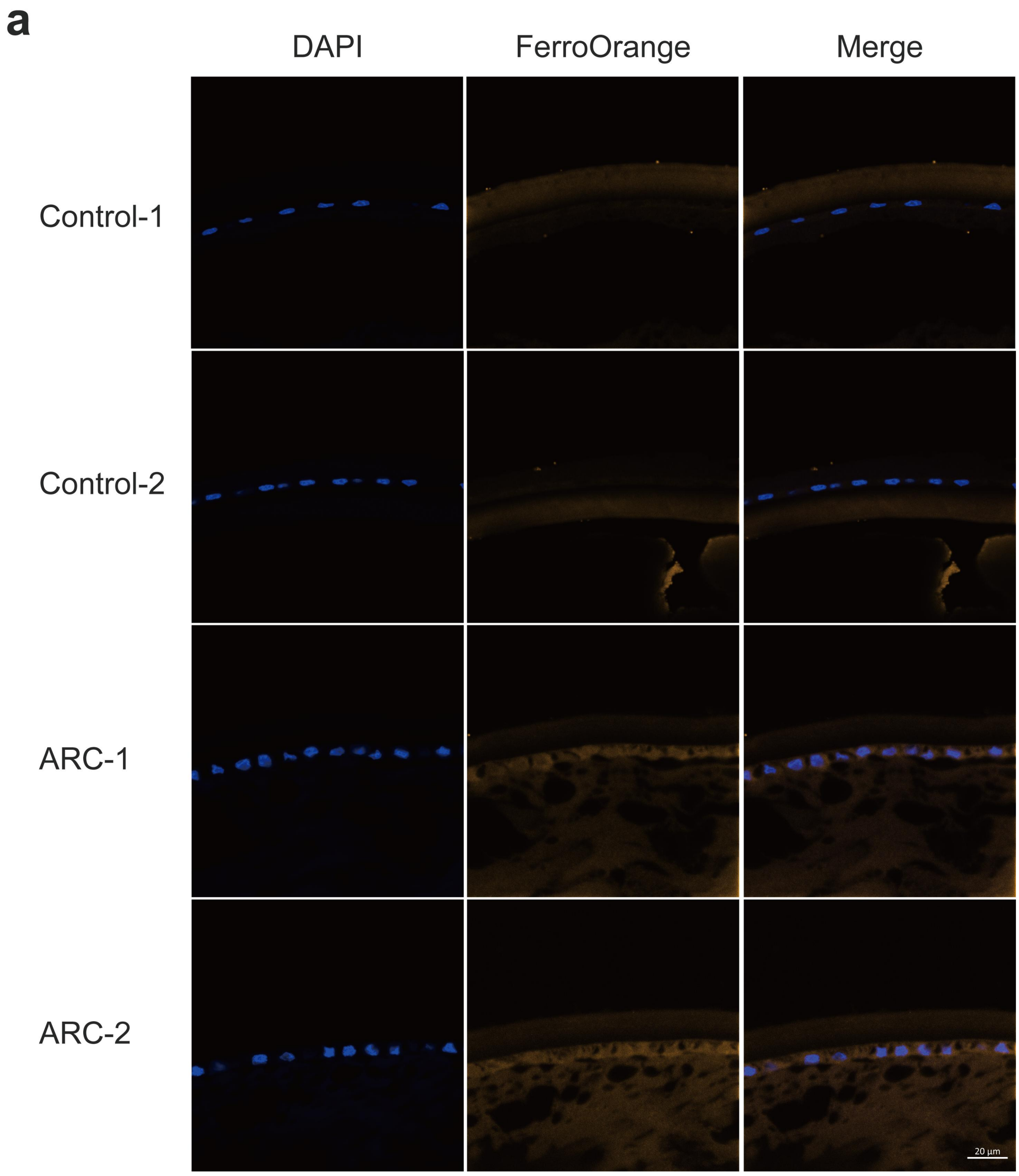

Supplement: Supplementary file 1 [file ijms-26-00307-s001.zip › ijms-3356547-supplementary.pdf]
